# Enhanced leaf nitrogen status stabilizes omnivore population density

**DOI:** 10.1007/s00442-016-3742-y

**Published:** 2016-10-07

**Authors:** Anna-Sara Liman, Peter Dalin, Christer Björkman

**Affiliations:** Department of Ecology, Swedish University of Agricultural Sciences, PO Box 7044, 750 07 Uppsala, Sweden

**Keywords:** Plant traits, Trophic omnivory, Population dynamics, Trophic status, Leaf nitrogen

## Abstract

Plant traits can mediate the strength of interactions between omnivorous predators and their prey through density effects and changes in the omnivores’ trophic behavior. In this study, we explored the established assumption that enhanced nutrient status in host plants strengthens the buffering effect of plant feeding for omnivorous predators, i.e., prevents rapid negative population growth during prey density decline and thereby increases and stabilizes omnivore population density. We analyzed 13 years of field data on population densities of a heteropteran omnivore on *Salix cinerea* stands, arranged along a measured leaf nitrogen gradient and found a 195 % increase in omnivore population density and a 63 % decrease in population variability with an increase in leaf nitrogen status from 26 to 40 mgN × g^−1^. We recreated the leaf nitrogen gradient in a greenhouse experiment and found, as expected, that increasing leaf nitrogen status enhanced omnivore performance but reduced per capita prey consumption. Feeding on high nitrogen status host plants can potentially decouple omnivore–prey population dynamics and allow omnivores to persist and function effectively at low prey densities to provide “background level” control of insect herbivores. This long-term effect is expected to outweigh the short-term effect on per capita prey consumption—resulting in a net increase in population predation rates with increasing leaf nitrogen status. Conservation biological control of insect pests that makes use of omnivore background control could, as a result, be manipulated via management of crop nitrogen status.

**Electronic supplementary material:**

The online version of this article (doi:10.1007/s00442-016-3742-y) contains supplementary material, which is available to authorized users.

## Introduction

Plant traits, such as nutrient status, morphology and secondary metabolites affect herbivore performance, long-term population dynamics and community structure and can even cascade across trophic levels to mediate trophic interactions (Price et al. 1980; Underwood and Rausher 2000; Harvey et al. 2003; Dalin and Björkman 2003; Kagata and Ohgushi 2006; Bukovinszky et al. 2008; Carmona et al. 2011; Volf et al. 2015). The interactions between plant-feeding omnivores and their herbivore prey can be mediated by plant traits (1) via density effects and (2) by altering the omnivores’ trophic status (behavioral effects) (Agrawal et al. 1999; Eubanks and Denno 1999, 2000; Eubanks 2005).

Omnivory and trophic behavior is determined by evolutionary history, driven by resource abundance and quality to optimize nutritional needs (Agrawal et al. 1999; Coll and Guershon 2002; Eubanks et al. 2003). Plant feeding allows for survival of small omnivore nymph stages and for persistence during periods of rapidly declining prey densities (Naranjo and Gibson 1996; Wheeler 2001; Coll and Guershon 2002; Eubanks and Styrsky 2005). Feeding on high-quality host plants can, thus, strengthen the buffering effect of this alternative resource, which should increase and stabilize omnivore population density and enhance the long-term predation pressure (density effect). High plant quality can, however, also increase the omnivores’ relative consumption of plant resources and reduce the average *per capita* predation rate (behavioral effect) (Eubanks and Denno 2000; Coll and Guershon 2002).

Nitrogen concentration is a characteristic of plant nutrient status with a potentially strong direct effect on omnivore performance in the absence of prey, which is also relevant for comparing the relative importance of resources at different trophic levels (Eubanks and Denno 1999, 2000; Fagan et al. 2002; Denno and Fagan 2003; Matsumura et al. 2004). Herbivore population density commonly increases on nitrogen enriched plants (Mattson 1980; Awmack and Leather 2002) and sap-feeding insects (i.e., many heteropteran omnivores) may be especially responsive to enhanced plant nitrogen, since they feed selectively and often on tissue that does not contain nitrogen-based allelochemicals (toxic secondary metabolites) (Holopainen et al. 1992; Wheeler 2001; Huberty and Denno 2004). Nitrogen concentration is substantially lower in plant than in herbivore biomass and the relative abundance of nitrogen in relation to growth conditions is, by orders of magnitude, more variable in plants than in herbivores (Mattson 1980; Sterner and Elser 2002; Andersen et al. 2004). The stoichiometric mismatch in nitrogen across trophic levels has also been critical for the evolution of omnivory in heteropteran insects (Eubanks et al. 2003).

In this study, we analyzed a 13-year time series on population density of an omnivorous heteropteran (*Orthotylus marginalis* Reuter, Miridae) in 17 gray willow (*Salix cinerea* L.) stands arranged along a measured leaf nitrogen gradient, with the aim of evaluating the established assumption that an increase in plant nutrient status (i.e., leaf nitrogen) increases and stabilizes omnivore population density. The leaf nitrogen gradient recorded in the field was recreated in a greenhouse, using cuttings from the willow clones in the field study, to test experimentally whether enhanced leaf nitrogen status increases omnivore performance and alters trophic behavior. More specifically, we expected: (1) omnivore population density and stability to increase with increasing host plant leaf nitrogen status; (2) omnivore performance to increase; and (3) omnivore predation rates to decrease with increasing leaf nitrogen status, since more access to plant nitrogen would reduce the need to consume prey.

## Methods

### Study system

The gray willow (*Salix cinerea* L.) grows in wet moderately nutrient-rich soils and often forms dense stands along small streams, ditches and pastures and at forest edges (Jonsell 2000). Stands generally consist of individuals of the same clone and range from a few square meters to several hectares in size (although small stands are more common).

Mirids (Heteroptera: Miridae) are among the most numerous insects on gray willow—both in terms of species and individuals (Strong et al. 1984). *Orthotylus marginalis* Reuter is often the most abundant species. This arboreal mirid is univoltine and overwinters as eggs inserted into crevices in the bark associated with leaf buds on the current year’s shoot (Kullenberg 1944). Nymphs emerge in late May and adults in early July. Mirids and many other heteropterans use a solid-to-liquid feeding method, i.e., they use salivary enzyme complexes to liquefy plant or prey tissues before eating them (Wheeler 2001). This adaptation allows for a very broad diet, i.e., mirids are able to make use of relatively large prey and to evaluate and access plant tissue of various qualities. Omnivorous mirids exhibit a wide range of phytophagous behaviors. *Orthotylus marginalis* is categorized as zoophytophagous, i.e., a primarily predatory omnivore that also utilizes, for example, the leaf veins that run through the mesophyll, which are generally rich in chlorophyll and influenced by nitrogen application (Kullenberg 1944; Wheeler 2001 and references therein).

Three leaf beetle species *Phratora vulgatissima* L., *Galerucella lineola* F. and *Lochmea caprea* L. (Coleroptera: Chrysomelidae) commonly reach outbreak densities in gray willow. *Orthotylus marginalis* frequently consumes the eggs of *P. vulgatissima* and young larvae of all species (Björkman et al. 2003, 2004). The generally high densities of willow leaf beetles suggest that they, at least temporarily, dominate as prey for these mirids. The mirids show behavioral numerical responses to the abundance of leaf beetles (Björkman et al. unpublished) and predation has population level effects on leaf beetle density and outbreak risk (Björkman et al. 2004; Dalin 2006).

### Leaf nitrogen gradients

Leaf nitrogen status in the field study and the greenhouse experiment was estimated using an optical chlorophyll meter (Model, SPAD-502, Konica Minolta Sensing, Japan). This is a non-destructive alternative to analytical methods that can be used to estimate leaf nitrogen content per unit area; the method is commonly used in plant sciences and has been extensively evaluated for a number of hardwoods, including *Salix* and *Populus* species (Bonneville and Fyles 2006; Weih and Rönnberg-Wästjung 2007; Bonosi et al. 2010). SPAD values are, however, mainly useful for relative comparisons of nitrogen content in leaves, under similar environmental conditions (Chang and Robison 2003).

SPAD values were recorded in 17 gray willow stands in May and June, 2011. The stands occupied two habitat types (open and forest edge) that were assumed to differ in soil nitrogen due to their different management practices (agriculture and forestry). SPAD recordings were made at five different positions between the mid-rib and the leaf margin on ten leaves in each stand. The leaves were selected to ensure that they were equally exposed to light, at the same vertical position above the ground as well as in the same position on the shoot (the third fully developed leaf). This sampling method should, therefore, avoid capturing within and between stand variation in leaf nitrogen status related to differences in vertical position on the tree, phenological status and light exposure. We repeated SPAD recordings in May and June to make sure that potential phenological differences between habitats did not in any way bias our recordings.

One-year-old shoots were cut from 15 of the gray willow stands and used to create a leaf nitrogen gradient in the greenhouse, similar to the one recorded in the field. The shoots were stored at −5 °C for 3 months and then divided into 90 (6 × 15) cuttings (length 20 cm, diameter 0.9–1.7 cm). The cuttings were placed in water in the greenhouse for 2 weeks to initiate root development, before being planted in pots with 2 dm^3^ sand (grain size 0.2–1 mm). The insides of the pots were lined with cloth, so that water and air (but not the sand) could pass through the holes in the base of the pots. The sand was saturated with water at all times. This ensured that water availability would not influence access to nitrogen and simulated the conditions gray willows experience in the field (growing in wet soils or more or less directly in water).

Six cuttings from each of the 15 stands were randomly assigned to three different fertilization treatments in the greenhouse, corresponding to 1.4, 8.4 or 15.4 mg N × week^−1^. These fertilization levels were used in a preceding pilot study and therefore known to create nitrogen levels similar to those observed in gray willow stands in the field. To fertilize the plants we used a complete nutrient solution, ‘Blomstra’ (Wallco, Sweden) (N:P:K 5:1:4.3, pH 7.8). The temperature in the greenhouse ranged from 18 to 24 °C. Leaf nitrogen status of plants in the greenhouse was recorded 30 days after the first fertilizer application to ensure that the selected treatments resulted in leaf nitrogen concentrations similar to those observed in the field. SPAD values were recorded at five different positions between the mid-rib and the leaf margin, on three leaves from the mid part of each plant.

The relationship between SPAD-502 chlorophyll meter values and leaf nitrogen concentrations was determined using three leaf samples collected from each gray willow stand (*n* = 51) in the field in June, 2011, covering almost the full spectrum of values found in gray willow stands at that time of the year. In addition, three leaf samples were collected from clones of the same willows grown in the greenhouse (*n* = 45). The aim was to determine whether the relationship between SPAD values and nitrogen concentrations differed between greenhouse and field samples, for instance due to differences in light and hydrological conditions. Mass-based leaf nitrogen concentrations of sampled leaves were determined by gas chromatography using a Carlo Erba NA 1500 Elemental Analyzer (Carlo Erba, Milano, Italy).

### Population density and variability

*Orthotylus marginalis* population density was estimated in 15 of the gray willow stands in June 1999–2011 and in the two other stands in 1999–2010 and 1999–2005. All insects on the upper 35 cm of each current-year shoot were dislodged into a white plastic container. *Orthotylus marginalis* nymphs were counted and then re-released. The number of shoots sampled was proportional to the size of the stand. For a detailed description of the sampling method see Dalin (2006).

### Performance and trophic behavior

Altogether 90 *O. marginalis* nymphs were collected for the greenhouse experiment from the same gray willow stand. All individuals were collected simultaneously, on the first day that first stage nymphs appeared in late May. The nymphs were randomly assigned to plants under different nitrogen treatments and plants with or without prey: 45 to plants with prey and 45 to plants without prey. Perforated plastic bags (0.5 mm diameter perforations, Baumann Saatzuchtbedarf, Germany) sealed at the base of the pot, prevented mirids from escaping and stopped potential prey from accessing the plants.

As prey for the nymphs, we added eggs of one of their common prey species, the willow leaf beetle *Phratora vulgatissima* (Coleoptera: Chrysomelidae) (Dalin 2006). Leaves with equally sized egg clutches were pinned to the underside of a leaf at the base of the plants. Further eggs were added every 48 h to ensure that there was always a surplus of eggs available to the nymphs. Omnivore performance was estimated by recording survival–non survival (broadly categorized as caused by parasitization or other factors), development time (number of days) and adult dry weight (mg). The total number of eggs consumed from first stage nymph to adult molting was divided by the number of development days and adult dry weight (number of eggs consumed day × mg^−1^) to eliminate the impact of differences between males and females.

### Statistics

The relationship between SPAD values and mass-based leaf nitrogen concentrations in leaves sampled in the field and in the greenhouse experiment was validated using a linear model. Leaf origin was treated as a fixed effect in the model.

The predicted difference in SPAD values between gray willow stands in the field study and nitrogen treatments in the greenhouse experiment was tested using two separate linear mixed models. Potential correlation between data points due to repeated measures of SPAD values in the field was accounted for by incorporating a first-order autoregressive correlation structure for the time variable (month). By treating stand as a random effect in the models, the intercept was allowed to vary among willow clones.

The relationship between *O. marginalis* population density and gray willow leaf nitrogen status in the field study was examined using a generalized linear mixed model (GLMM) (Poisson distribution, log link function) with an offset for number of samples in each stand. The fixed effects were the SPAD values recorded in June, sampling year and stand area. By treating gray willow stand as a random effect, the model intercept was allowed to vary between stands and between years within stands. Temporal autocorrelation was accounted for by introducing an autoregressive first-order structure, with observation year nested within willow stand.

Two measures were used to estimate population variability: the coefficient of variation (CV) and the inter-quartile ratio (IQR). CV was calculated for each stand by dividing the standard deviation of density by the mean density. CV thus provides a standardized measure of variability which allows for comparisons regardless of the mean. However, CV is sensitive to zero counts and low means (Heath 2006). The IQR equals the inter-quartile range divided by the median and provides an alternative measure of variability that is independent of both observation extremes and the mean. We fitted two separate linear models with the response variable population variability expressed either as CV or IQR and leaf nitrogen status and willow stand area as fixed effects. CV and IQR were estimated using a subset of the population density data (for 15 stands, 1999–2008). The selection of data was intended to optimize the number of stands sampled over the same years and the longest continuous time period. The model was restricted to SPAD values in the range 30–36 because of the low number of observations below this range (SPAD < 30). Including or excluding the two observations in this lower range in the model did not change the overall results.

The effects of leaf nitrogen status on performance and predation rate in the greenhouse experiment were examined using two GLMMs. Response variables in the two performance models were *O. marginalis* survival (binomial distribution, logit link) and adult dry weight (µg) (Poisson distribution, log link). The response variable in the predation model was total egg consumption by *O. marginalis* (Poisson distribution, log link) using an offset for development time (days) and adult dry weight (mg). Over-dispersion was accounted for using quasi-models. Fixed effects in both performance models were nitrogen treatment and the presence/absence of prey, and in the predation model nitrogen treatment. We incorporated a variance structure in the omnivore performance models to account for differences in residual spread between groups with and without prey. Gray willow clone was included as a random effect in all models.

Analyses were performed in R version 3.1.0 (R Development Core Team 2014) using the MASS package glmmPQL function for the Poisson GLMMs (Venables and Ripley 2002) and the nlme package lme function for the linear mixed model (Pinheiro et al. 2011).

## Results

### Leaf nitrogen gradients

**Fig. 1:**
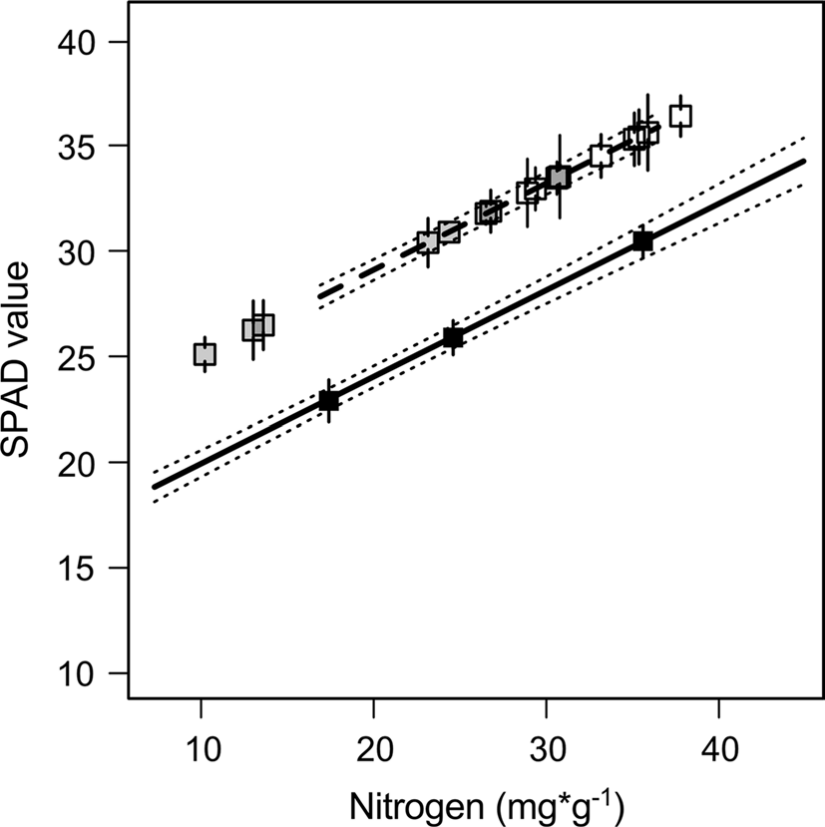
SPAD-leaf nitrogen validation models plotted with field- and greenhouse-recorded leaf nitrogen gradients. Predictions were based on linear models describing the relationship between SPAD values and leaf nitrogen concentrations (mg × g^−1^) in gray willow leaves (*R* ^2^ = 0.67), collected in the greenhouse (*solid line*) and the field (*dotted line*). Fine *dotted gray lines* are standard errors of prediction estimates. *Open squares* show mean SPAD values recorded in June for 17 gray willow (*Salix cinerea*) stands in forest and open habitats (*squares* with *white* and *gray* backgrounds, respectively). *Solid squares* show mean greenhouse SPAD values recorded under the 1.4, 8.4 and 15.4 mg N × week^−1^ nitrogen treatments. *Bars* show standard errors

There was a positive relationship between recorded SPAD values and mass-based nitrogen concentrations (mg × g)^−1^ (*F*_1,93_ = 111.04, *P* < 0.001, *R* ^2^ = 0.67, Fig. 1). The model intercept varied between leaves collected from the field and those in the greenhouse (*F*_1,93_ = 51.67, *P* < 0.001, Fig. 1). The relationship was best described by the model SPAD = 0.41N_m_ + 15.83 × greenhouse + 5.07 × field (Fig. 1).

The recorded SPAD values ranged from 25.07 to 36.39 in May and from 20.06 to 36.72 in June (Fig. 1). SPAD values for leaves from the 17 gray willow stands were significantly different (*F*_1,15_ = 53.54, *P* < 0.001). The May and June gradients were similar with respect to their intercept (*F*_1,148_ = 1.50, *P* = 0.22) and slope (*F*_1,148_ = 0.30, *P* = 0.58). SPAD values recorded in the greenhouse experiment were higher for plants under higher nitrogen treatments (*F*_2,118_ = 41.25, *P* < 0.001). When SPAD values were translated into nitrogen concentrations using the SPAD–nitrogen validation for gray willow in the greenhouse, the range was similar to the field-recorded nitrogen concentrations (Fig. 1).

### Population density and variability

**Fig. 2:**
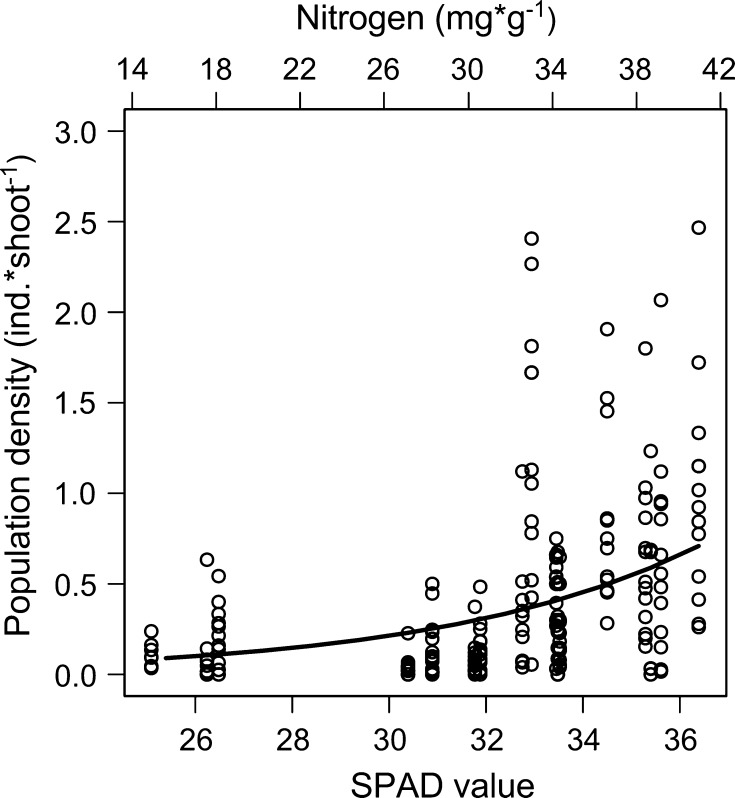
Population densities related to leaf nitrogen status. Predicted mean number of *Orthotylus marginalis* individuals per 35 cm shoot (based on data from 17 gray willow stands collected over 13 years) related to measured SPAD values (relative difference in leaf nitrogen concentration—*lower horizontal scale*) and to the predicted corresponding leaf nitrogen concentration (*upper horizontal scale*)

There was a positive relationship between mirid omnivore population density and leaf nitrogen status of the gray willow stands in the field (*F*_1,14_ = 10.67, *P* = 0.006, Fig. 2). Population density increased by 195 % with a SPAD value increase from 30.75 to 36.49, which is approximately equivalent to an average increase in leaf nitrogen from 26 to 40 mgN × g^−1^(Fig. 2). Population density also differed between sampling years (*F*_12,174_ = 7.63, *P* < 0.001), but there was no effect of willow stand area (*F*_1,14_ = 0.002, *P* = 0.97).

**Fig. 3:**
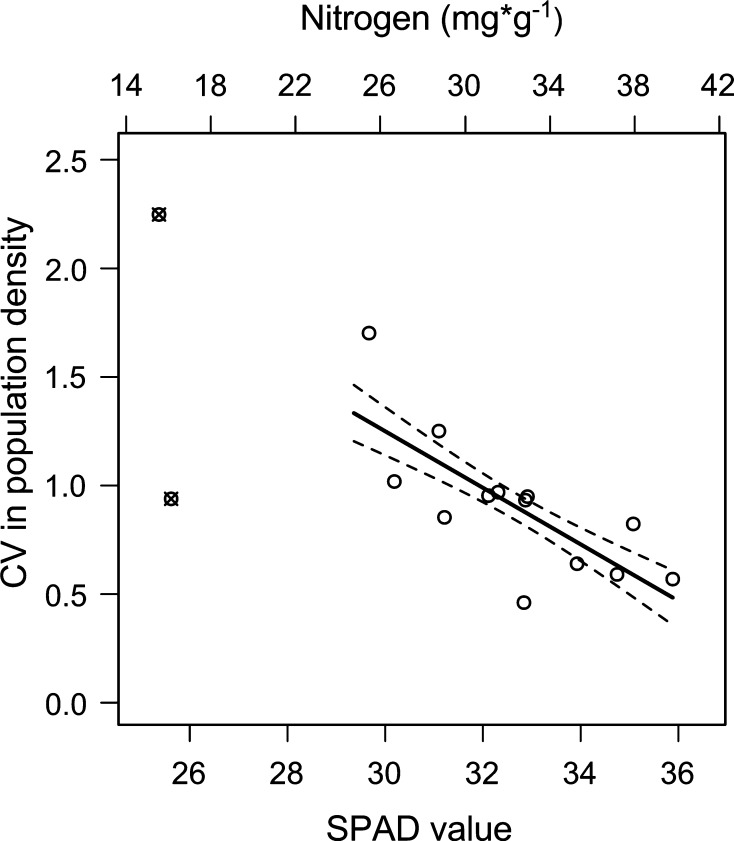
Population variability related to leaf nitrogen status. Variability (CV = coefficient of variation) in population density of *Orthotylus marginalis* related to measured SPAD values (relative difference in leaf nitrogen concentration—*lower horizontal scale*) and to the predicted corresponding leaf nitrogen concentration (*upper horizontal scale*). *Solid* and *dotted lines* indicate model predictions and standard error of predicted means (*R* ^2^ = 0.53), respectively. *Open circles* are data points used in the model. Data points indicated by crosses were not considered in the model. Population variability was calculated from data collected in 15 stands over 10 years. A small CV represents high stability between years

*Orthotylus marginalis* populations in relatively nitrogen-rich stands varied less between years than populations in more nitrogen-poor stands (CV: *F*_1,10_ = 14.41, *P* = 0.004, *R* ^2^ = 0.56 and IQR: *F*_1,10_ = 9.86, *P* = 0.01, *R* ^2^ = 0.44). Population variability, expressed as CV, decreased by 63 % with a SPAD value increase from 30.75 to 36.49, which is approximately equivalent to an average increase in leaf nitrogen from 26 to 40 mgN × g^−1^(Fig. 3). Willow stand area did not explain differences in population variability (CV: *F*_1,10_ = 0.96, *P* = 0.35 and IQR: *F*_1,10_ = 1.11, *P* = 0.32). The two measures of population variability were correlated (Pearson correlation, *r* = 0.52, *P* = 0.048).

### Performance and trophic behavior

Total numbers of *O. marginalis* nymphs surviving to the adult stage under the 1.4, 8.4 and 15.4 mg N week^−1^ nitrogen treatments were: 6 in all cases with prey absent; and 15, 10 and 13, respectively, with prey present. *Orthotylus marginalis* survival was clearly higher on plants in the presence of prey (*F*_1,72_ = 22.41, *P* < 0.001), but there was no difference in survival on plants under different nitrogen treatments (*F*_2,72_ = 1.50, *P* = 0.23). However, these results should be treated cautiously as various mortality factors, such as parasitization and molting failure, were not necessarily related to leaf nitrogen status. Mortality due to parasitization was 16 % for *O. marginalis* with no prey and 11 % with prey present.

**Fig. 4:**
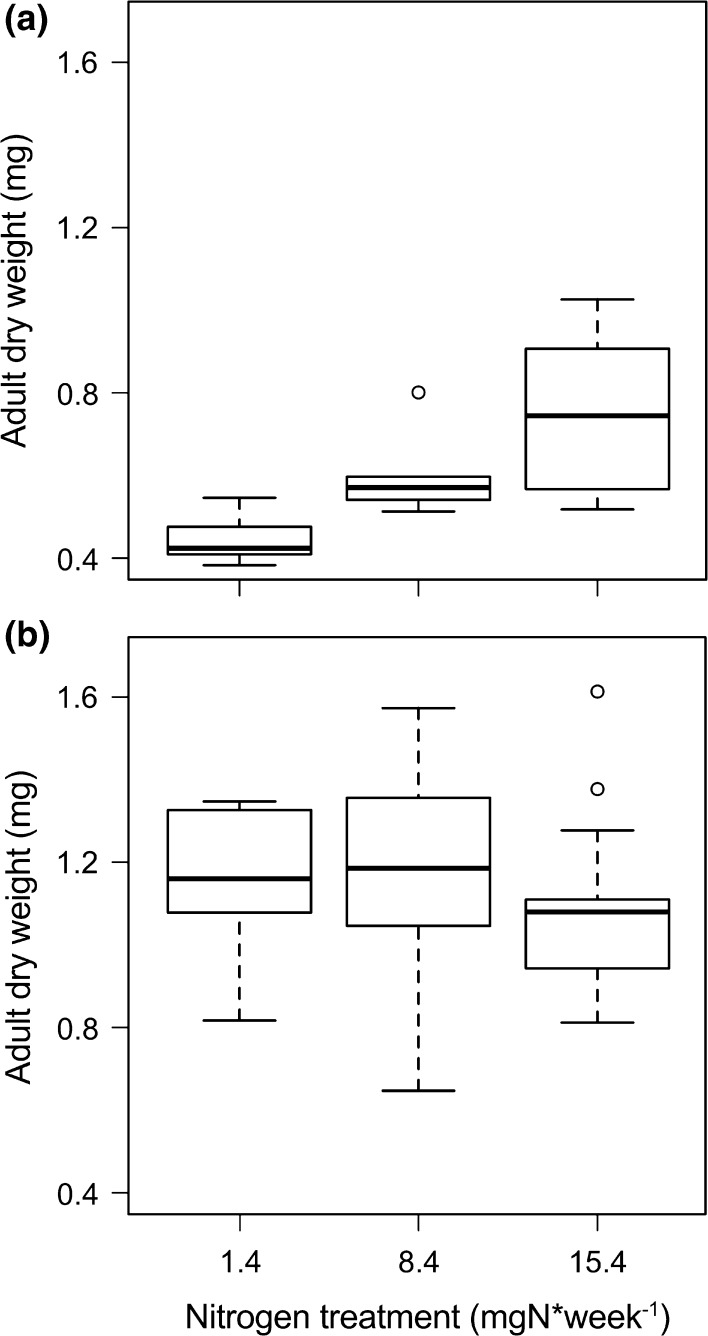
Performance related to leaf nitrogen status. Dry weight (mg) of *Orthotylus marginalis* adults in the **a** absence (*n* = 18) and **b** presence of prey (*n* = 38) on plants exposed to different nitrogen treatments. Performance differed between all nitrogen treatments in the absence of prey at *P* < 0.01, but there was no difference between any of the treatments in the presence of prey. *Box plots* show medians with the first and third quartile and 95 % confidence interval of the median

Adult dry weight was generally higher when prey were present on the host plant (*F*_1,36_ = 51.73, *P* < 0.001) and higher on plants exposed to higher nitrogen treatments *F*_1,36_ = 5.76, *P* = 0.007, but only in the absence of prey (*F*_2,36_ = 5.95, *P* = 0.006, Fig. 4). Adults with access to both plant and prey exhibited, on average, 89 % higher dry weight, compared to individuals without access to prey. Dry weight of individuals without prey increased by, on average, 69 % from the lowest (1.4 mg N week^−1^) to the highest (15.4 mg N week^−1^) plant nitrogen treatment.

**Fig. 5:**
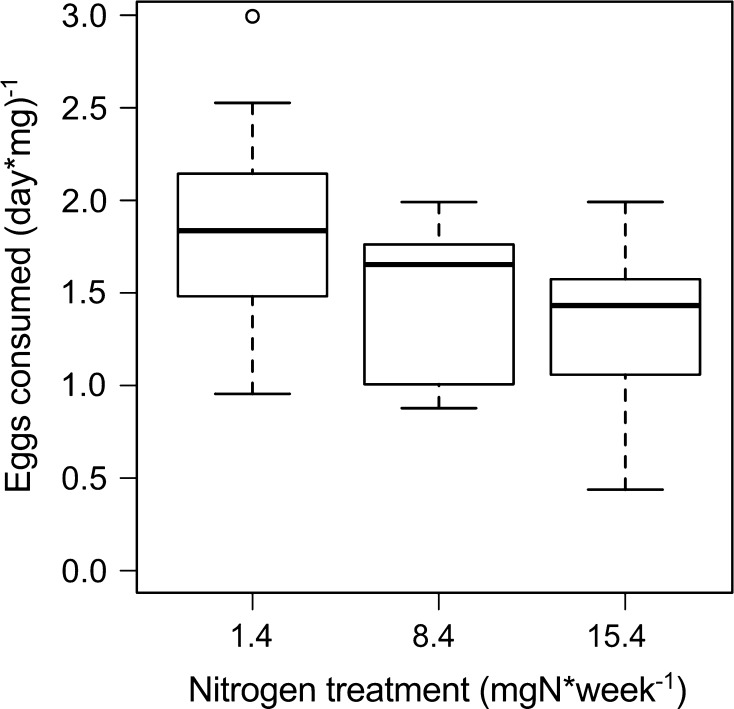
Predation rate related to leaf nitrogen status. Number of leaf beetle eggs consumed by *Orthotylus marginalis* (day × mg dry weight)^−1^ related to leaf nitrogen status under three nitrogen treatment levels (*n* = 37). Predation rates differed between all treatments at *P* < 0.01. *Box plots* show medians with the first and third quartile and 95 % confidence interval of the median

The number of eggs consumed (day × mg^−1^) was lower on plants receiving higher nitrogen applications (*F*_2,20_ = 6.75, *P* = 0.006, Fig. 5) and differed between all treatments at *P* < 0.01. Predation rate decreased by, on average, 28 % from the lowest (1.4 mg N week^−1^) to the highest (15.4 mg N week^−1^) plant nitrogen treatment.

## Discussion

We utilized a combination of data from long-term field studies and controlled greenhouse experiments to show that feeding on plant resources with a high nitrogen status increases and stabilizes omnivore population density, and enhances performance in the absence of prey. To our knowledge, this is the first empirical evidence under field conditions for this effect. Moreover, we found that leaf nitrogen status can alter the omnivore’s trophic behavior: individuals on plants with higher nitrogen status consume less prey. Both results are consistent with accumulating (but scattered) evidence linking the distribution, dispersal, performance, oviposition preference and trophic behavior of omnivorous insects to intra-specific variation in plant quality (Agrawal et al. 1999; Eubanks and Denno 1999, 2000; Eubanks and Styrsky 2005; Groenteman et al. 2006; Jiménez et al. 2012).

High plant nitrogen concentration can enhance performance and alter the trophic behavior of these (and other) omnivores via the nutritional value of plant tissue—but it could also operate via a variety of associated plant traits. Leaf nitrogen can, e.g., reflect the carbon-to-nitrogen ratio and the accumulation of secondary metabolites (Koricheva et al. 1998). Allelochemicals may affect heteropteran performance and trophic behavior, but studies targeting this group are still rare and results generally inconsistent (Kaplan and Thaler 2011). Plant nitrogen status may also indicate availability and abundance of limiting resources other than plant nutrients. Variation in willow shoot growth may, for example affect the inter- and intra-specific competition among mirids for suitable oviposition sites—since oviposition is limited to growing tissue in many arboreal mirids (Wheeler 2001 and references therein).

Leaf nitrogen status could also indirectly affect the spatial and temporal variation in omnivore population density—via variation in prey abundance. Leaf beetles are among the most numerous herbivorous insects and their eggs and larvae dominate as prey for *O. marginalis* and other heteropteran predators on gray willow (Björkman et al. 2003; Dalin 2006). The relative importance of direct (plant quality) and indirect (prey availability) effects of plant nitrogen status for this omnivore remains to be explored experimentally. However, two studies involving this system suggest that omnivore population density is more tightly associated with variations in plant quality than variations in prey density (Dalin 2006, Liman et al. unpublished). These studies indicate that leaf beetle performance in the field does not increase with increasing leaf nitrogen status and high predation pressure from omnivorous mirids limits leaf beetle population densities in nitrogen-rich habitats. Furthermore, analysis of long-term field data, shows that the mirids do not respond numerically to leaf beetle population growth rates—which implies that plant feeding may uncouple omnivore–prey population dynamics.

The long-term population level effect and the behavioral effect (plant vs. prey feeding) of leaf nitrogen status differed with respect to the consequence for omnivore prey suppression and the outcome of the plant–herbivore–omnivore interaction. The different environmental conditions (field vs. greenhouse) and spatial and temporal scales make it impossible to integrate the effects entirely and to predict the net outcome of leaf nitrogen status on prey suppression. The density effect associated with increased plant nutrient status seems, however, as expected, to be stronger than the per capita changes in trophic behavior—especially when longer time scales are considered **(**Eubanks and Denno 2000). Consequently, we expect the long-term effect of high leaf nitrogen status (population stability) to outweigh the short-term behavioral effect on per capita predation rates—resulting in a net increase in population predation rates with increasing leaf nitrogen status.

Trophic omnivores alternating between plant and prey include, by definition, a wide range of phytophagous behaviors, from primarily herbivorous to primarily predatory and the feeding strategies of mirids and other omnivorous heteropteran predators can, apart from plant feeding, also include additional trophic interactions such as intraguild predation, cannibalism and predatory scavenging (Rosenheim and Corbett 2003; Law and Rosenheim 2011; Krimmel and Pearse 2013). The prevalence of omnivory and “weak” direct interactions between species in a food web has been shown to be important for our understanding of food web dynamics and the persistence and stability of ecosystems (Emmerson and Yearsley 2004; Vandermeer 2006; Kratina et al. 2012). Empirical studies on the role of omnivory for stability at the population and community level (such as the present study) can contribute to a better understanding of patterns observed at food web level.

## Conclusion

This study provides long-term field data and greenhouse experimental data to show that leaf nitrogen status can affect the stability of omnivore populations and alter individual trophic behavior. Plant nutrient status may, therefore, also determine the degree of coupling between omnivores and their prey populations, which will have consequences for when predators are functionally effective (i.e., when predator-to-prey ratios are high). Omnivores can (if they are decoupled from prey density) persist and function effectively at low prey densities (unlike specialist predators) to provide what has been referred to as ‘background level’ control of insect pests (Symondson et al. 2002). Conservation biological control of insect pests utilizing omnivore “background level” control could, as a result, be modified via crop properties, e.g., by maintaining a continuous, high-quality host plant resource or by selecting for plant traits that optimize omnivore density effects.

## Electronic supplementary material

Below is the link to the electronic supplementary material.

Supplementary material 1 (DOCX 348 kb)

Supplementary material 2 (DOCX 11 kb)

Supplementary material 3 (CSV 1 kb)

Supplementary material 4 (CSV 8 kb)

Supplementary material 5 (CSV 3 kb)
