# Supplementary material for: Enhanced leaf nitrogen status stabilizes omnivore population density
Source: Oecologia. 2016 Oct 7;183(1):57–65. doi: 10.1007/s00442-016-3742-y (PMC5239808; doi:10.1007/s00442-016-3742-y)
Supplement: Supplementary file 1 — Supplementary material 1 (DOCX 348 kb) [file 442_2016_3742_MOESM1_ESM.docx]

**Appendix S1**


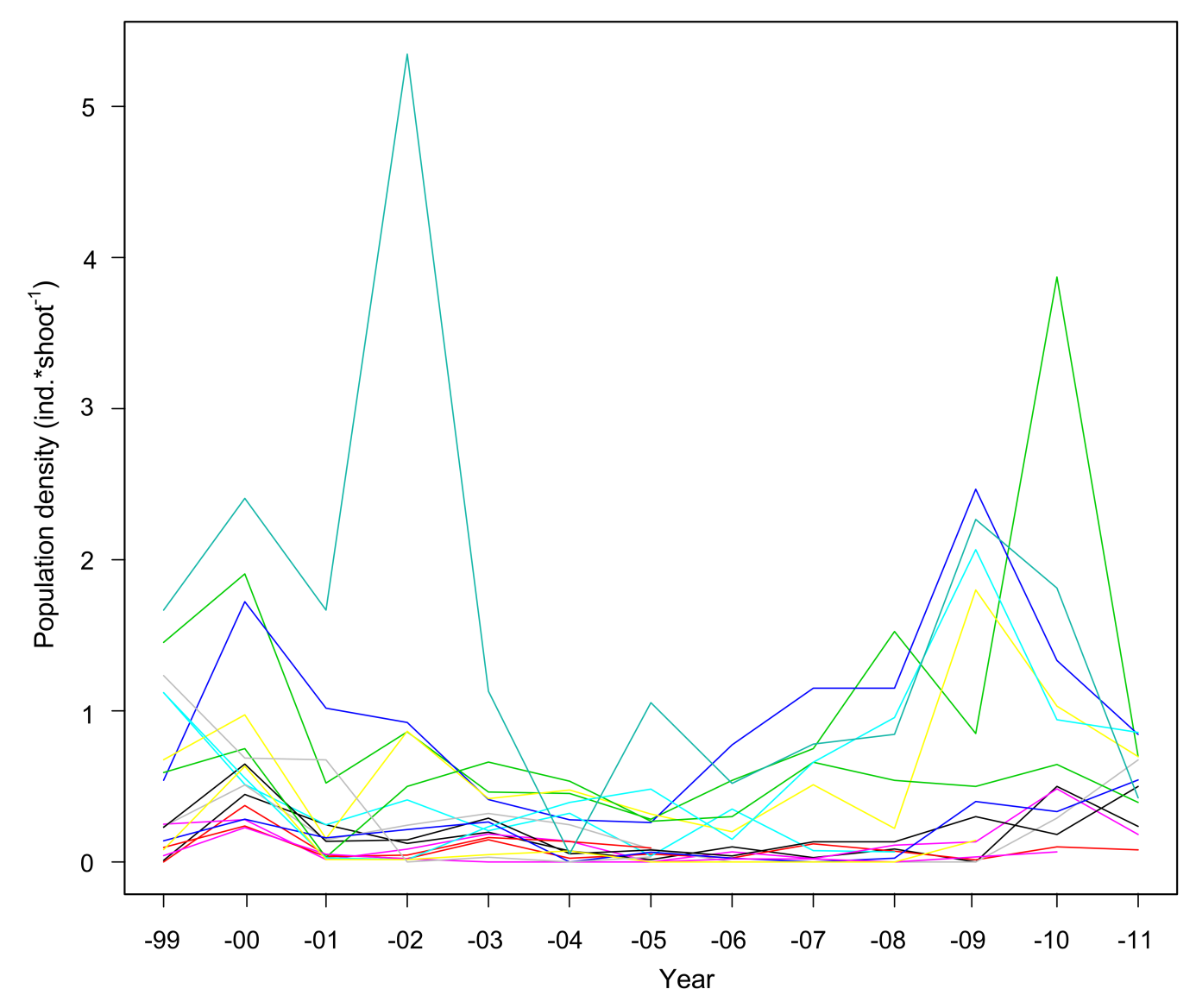
**Fig. 1** Population density of the omnivorous predator *Orthotylus marginalis* over 13 years. Lines (unique colours) show the number of individuals per 35 cm shoot in 15 grey willow stands 1999-2011 and in two stands 1999-2010 and 1999-2005 respectively. Note that three of the lines (with mean values below 0.5 ind*shoot^-1^) are partly hidden by other lines
