# Supplementary material for: Enhanced leaf nitrogen status stabilizes omnivore population density
Source: Oecologia. 2016 Oct 7;183(1):57–65. doi: 10.1007/s00442-016-3742-y (PMC5239808; doi:10.1007/s00442-016-3742-y)
Supplement: Supplementary file 2 — Supplementary material 2 (DOCX 11 kb) [file 442_2016_3742_MOESM2_ESM.docx]

#Metadata "S2_Dataset.csv" Data used to validate the relationship between leaf nitrogen concentration and SPAD values (Figure 1)

id grey willow stand id

obs repeated observations within stand (1,2,3)

type leaf origin (1=field, 2= greenhouse)

spad SPAD-value (leaf average)

N nitrogen concentration (mg/g)

#Metadata S3_Dataset.csv *Orthotylus marginalis* population density and variability and leaf nitrogen status (Figures 2 and 3 and Figure S1).

id grey willow stand id

lat site latitude (WGS84)

lon site longitude (WGS84)

area grey willow stand area (ha)

year observation year

spad.june SPAD value, stand average june 2011

count number of individuals per stand

samples number of samples per stand

(population density (ind*sample^-1^)= counts/samples)

#Metadata "S4_Dataset.csv" *Orthotylus marginalis* performance and predation rates and leaf nitrogen status (Figures 4 and 5)

id grey willow stand id

prey prey treatment (1=prey absent, 2=prey present)

fert N treatment 1=1.4, 2=8.4, 3=15.4 (mgN/week)

spad-value SPAD recording

surv 0=alive, 1=dead

mort.factor 0=unknown, 1=parasite

sex 1=female, 2=male

devdays number of development days

weight(mg) insect adult dry weight (mg)

egg.pred total number of eggs predated
